# Supplementary material for: Complexome profiling of the Chlamydomonas psb28 mutant reveals TEF5 as an early PSII assembly factor
Source: Plant Cell. 2025 Mar 17;37(6):koaf055. doi: 10.1093/plcell/koaf055 (PMC12164590; doi:10.1093/plcell/koaf055)
Supplement: koaf055_Supplementary_Data [file koaf055_supplementary_data.zip › psb28_interactive.zip!/plots/intensity/qualityPlots/peptides/LHCB4_Cre17.g720250.t1.2.html]

### Cre17.g720250.t1.2

Trivial name: LHCB4  
SeqCoverage [median]: 72.90  
Mass: 29.94 kDa  
  
 

| Sequence | MissedCleavage | Mass [Da] | wtIntensity | muIntensity | Score |
| --- | --- | --- | --- | --- | --- |
| AGTTATKPAPK | 1 | 1041.58 | 493443.46 | 250808.18 | 241.13 |
| FADGLNNGK | 0 | 934.45 | 407866.18 | 370930.72 | 209.84 |
| FADGLNNGKGL | 1 | 1104.56 | 4589.62 | 3396.91 | 126.07 |
| FPTPPGTQK | 0 | 971.51 | 882905.43 | 486729.90 | 211.39 |
| FPTPPGTQKK | 1 | 1099.60 | 151480.83 | 42950.78 | 160.75 |
| FRECELIHGR | 1 | 1258.62 | 11680.15 | 0.00 | 42.00 |
| GFDPLGLSK | 0 | 932.50 | 56948.77 | 25305.92 | 134.05 |
| GFDPLGLSKPSEFVVIGVDENDQNAAK | 1 | 2845.41 | 207525.38 | 126860.60 | 150.91 |
| GSVEAIVQATPDEVSSENR | 0 | 1986.95 | 1962546.64 | 1441288.24 | 343.96 |
| KAGTTATKPAPK | 2 | 1169.68 | 2317.12 | 3433.49 | 156.36 |
| KVATSTGTR | 1 | 919.51 | 730.71 | 908.74 | 81.64 |
| KYQGDALWLPNTTRPEWLDGSLPGDR | 2 | 2984.48 | 76056.29 | 0.00 | 89.56 |
| LAMVSFFGYGVQALSTGEGALGSLAK | 0 | 2573.32 | 120322.11 | 28989.73 | 161.04 |
| LAPYSEVFGLAR | 0 | 1321.70 | 3289729.48 | 2160762.91 | 297.28 |
| LASEDEER | 0 | 947.42 | 2007914.67 | 911093.37 | 254.37 |
| LASEDEERAFR | 1 | 1321.63 | 84840.36 | 44777.22 | 147.37 |
| LKTAEIK | 1 | 801.50 | 102511.49 | 89636.37 | 147.52 |
| LKTAEIKHAR | 2 | 1165.69 | 0.00 | 1073.31 | 104.82 |
| NNKGSVEAIVQATPDEVSSENR | 1 | 2343.13 | 522227.48 | 297119.96 | 192.09 |
| NSETNPEK | 0 | 917.41 | 2985.49 | 5498.77 | 172.25 |
| NSETNPEKR | 1 | 1073.51 | 88272.85 | 97860.93 | 127.76 |
| PSEFVVIGVDENDQNAAK | 0 | 1930.93 | 21839.12 | 18669.33 | 164.48 |
| SGGVGYRK | 1 | 822.43 | 170859.86 | 51789.76 | 182.95 |
| VATSTGTR | 0 | 791.41 | 643144.44 | 380075.38 | 169.97 |
| VFKFPTPPGTQK | 1 | 1345.74 | 644940.98 | 408726.94 | 231.54 |
| VFKFPTPPGTQKK | 2 | 1473.83 | 31287.88 | 31650.86 | 78.49 |
| YQGDALWLPNTTRPEWLDGSLPGDR | 1 | 2856.38 | 11654.76 | 0.00 | 120.33 |
|  |  |  | 12000621.6 | 7280338.3 |  |
